# Microbial Genomics: Innovative Targets and Mechanisms

**DOI:** 10.3390/antibiotics12020190

**Published:** 2023-01-17

**Authors:** Asma Hussain Alkatheri, Polly Soo-Xi Yap, Aisha Abushelaibi, Kok-Song Lai, Wan-Hee Cheng, Swee-Hua Erin Lim

**Affiliations:** 1 Health Sciences Division, Abu Dhabi Women’s College, Higher Colleges of Technology, Abu Dhabi 41012, United Arab Emirates; 2 Jeffrey Cheah School of Medicine and Health Sciences, Monash University Malaysia, Jalan Lagoon Selatan, Bandar Sunway, Selangor 47500, Malaysia; 3 Office of Campus Director, Abu Dhabi Colleges, Higher Colleges of Technology, Abu Dhabi 41012, United Arab Emirates; 4 Faculty of Health and Life Sciences, INTI International University, Persiaran Perdana BBN, Nilai 71800, Malaysia

**Keywords:** antibiotic resistance, genomics, novel antibiotics, whole genome sequence, biosynthetic gene clusters, virulence factors

## Abstract

Multidrug resistance (MDR) has become an increasing threat to global health because bacteria can develop resistance to antibiotics over time. Scientists worldwide are searching for new approaches that go beyond traditional antibiotic discovery and development pipelines. Advances in genomics, however, opened up an unexplored therapeutic opportunity for the discovery of new antibacterial agents. Genomic approaches have been used to discover several novel antibiotics that target critical processes for bacterial growth and survival, including histidine kinases (HKs), LpxC, FabI, peptide deformylase (PDF), and aminoacyl-tRNA synthetases (AaRS). In this review, we will discuss the use of microbial genomics in the search for innovative and promising drug targets as well as the mechanisms of action for novel antimicrobial agents. We will also discuss future directions on how the utilization of the microbial genomics approach could improve the odds of antibiotic development having a more successful outcome.

## 1. Introduction

**Figure 1:**
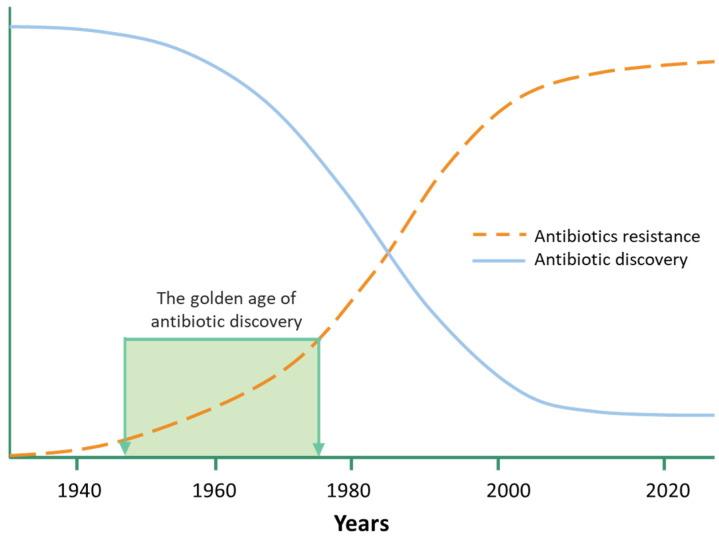
Discovery of antibiotics vs. development of resistance. The golden age for the discovery of antibiotics was between 1945–1975. After that, the discovery of antibiotics began to decline significantly. The main reason behind this is the increase in the development of antibiotic resistance in most pathogens, making the drugs ineffective against infections caused by MDR bacteria.

Fifty years after the introduction of antibiotics as therapeutic agents, there are now only a few effective antibiotics for serious bacterial infections due to the development of resistance against the available drugs [1]. In the past 20 years, we have witnessed a noticeable decline in the discovery of new antibiotics, and with that, the escalation of resistance against the antibiotics now being marketed at an alarming rate as shown in Figure 1. Consequently, multidrug resistance (MDR) has become widespread amongst various microbial species throughout the world [2,3]. Based on what was published by the US Centers for Disease Control and Prevention in a “threat report”, it is estimated that two million adults develop antibiotic-resistant infections every year in the United States, resulting in 23,000 deaths [3,4].

After the sequencing of the first complete genome of the bacterium was completed, there was a glimmer of hope for the treatment of infections caused by multidrug-resistant bacteria after conventional approaches had failed [5]. Several pharmaceutical companies have returned to the antibiotic market, which increases efforts to discover antibacterial agents using genomics, by finding innovative targets and mechanisms [3]. Almost two decades later, in the post-genomics era, many companies have identified innovative and effective targets for the development of new antibiotics. For instance, bioactive metabolic pathways in bacterial cells have been exploited as drug targets, such as two-component signal transduction systems (TCSTS) and histidine kinases (HKs) [6,7].

In addition, the bacterial genomics approach has been used to identify many drugs that target fundamental processes of bacterial cell growth. Several targets have been identified by using different strategies for bacterial genomes, including LpxC, FabI, peptide deformylase (PDF), and aminoacyl-tRNA synthetases, which are discussed in this review [8].

## 2. The Microbial Genomes Contribute to the Discovery of Novel Antimicrobial Agents

Knowledge of the genome sequence of bacteria in conjunction with bioinformatics approaches provides the opportunity to gather information about the major genes that contribute to the pathogens causing disease as well as their importance and the extent of their expression during infection. These are attractive targets for the development of antibiotics [3,9]. In 1995, the first whole-genome sequence was discovered for *Haemophilus influenzae*. Later that year, the second whole-genome sequence was discovered for *Mycoplasma genitalium*, which is much smaller than the first whole-genome sequence. Knowledge of two different whole-genome sequences allowed detailed study of their differences. This led to the emergence of the concept of the “minimal gene set for cellular life”, which is concerned with predicting the minimum number of genes required for bacterial survival [10]. Currently, more than 130,000 complete and near-complete genome sequences are available, with more than 376,000 genome projects around the world deposited and provided in public databases [11,12].

Comparative genomic studies have shown that the bacterial genome is a dynamic entity shaped by various forces, including genome shrinkage, loss or duplication of genes, genome restructuring, and lateral gene transfer to acquire new genes [13]. It can be concluded that knowledge of the genome sequence of a single strain is not sufficient to fully understand the genetic composition of human pathogens. For example, the results of comparing the genetic repertoire of a large number of pathogenic strains of *Helicobacter pylori* isolated from one patient showed that a single clone colonizing a specific environment such as the stomach can give rise to considerable genetic diversity [14,15]. Another example is the fact that the genetic sequence of *E. coli* O157:H7 contains more than 1300 strain-specific genes compared to the genetic sequence of the *E. coli* laboratory strain K12 [16].

Genome sequencing helps identify genes involved in the biological interactions of bacteria and contributes to the formation of the physiological and structural components of bacteria. Bacterial genomics is the most effective technology available to improve yield, efficacy, and success rate in the development of new antibacterial agents. Moreover, understanding genomics is not limited to identifying innovative targets for novel antibiotics. It also provides the opportunity to understand the fundamental interactions involved in using antibacterial agents as therapeutics. In addition, it has led to obtaining data about the genes responsible for several important processes, such as bacterial adaptation, host vitality, pharmacokinetics, and multidrug resistance, as well as metabolic processes that help in understanding secondary metabolites, nitrotoluene degradation, and lipopolysaccharide biosynthesis [17,18].

Using genome sequencing for a range of pathogens provides the opportunity to compare all genes to determine which genes are shared between these species [19]. Tatusov and his partners suggested that genes present in bacteria but absent in eukaryotes might be ideal targets for the development of broad-spectrum antibiotics [20]. Indeed, Mushegian and Koonin identified 256 common genes in the complete genome sequences of *H. influenzae* and *M. genitalium* [21]. In addition, Arigoni et al. identified 26 genes in *Escherichia coli*, most of which are conserved in the genomes of *Bacillus subtilis*, *M.genitalium, H. influenzae*, *H. pylori, Streptococcus pneumoniae*, and *Borrelia burgdorferi*. So, this list of genes may contain novel targets for antibiotic development. In addition, the selectivity of these targets can be studied by comparing them to the genomes of eukaryotes [22]. The more genome sequences of bacteria are known, the greater the chance of discovering innovative targets for the development of antimicrobial agents.

## 3. Pharmaceutical Companies’ Strategies for Using Bacterial Genomics to Discover Novel Antibacterial Agents

**Figure 2:**
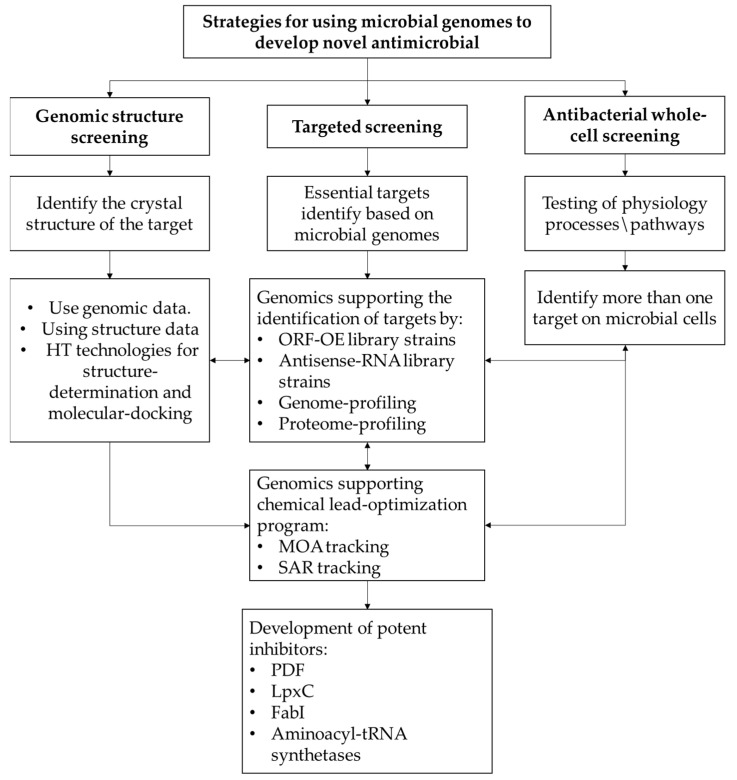
Flowchart of different strategies of using genomics to discover antibacterial.

Post-genomics will be the new golden age for the discovery of antibiotics. Pharmaceutical companies have developed several strategies to use genomics to discover innovative and effective antibiotics, namely targeted screening, genomic structure screening, and whole-cell antibacterial screening as shown in Figure 2 [5].

**Strategy i:** Target-based screening.

Genomics has contributed significantly to identifying a large number of attractive, diverse, and essential targets in the bacterial cell life cycle. This strategy is favored by several pharmaceutical companies, including Eli Lilly, Genome Therapeutics, GlaxoSmithKline, Johnson & Johnson, Merck, Millennium, Novartis, Oscient, and Pfizer. This strategy has reported four innovative and potent in vitro targets (PDF, LpxC, FabI, and aminoacyl-tRNA synthetases) mentioned below. This strategy is proving successful in identifying new targets without clinical resistance, knowledge of the mode of action (MOA) of antibiotics, and enzyme hits that do not need to enter the bacterial cell [5,23,24,25,26,27,28].

**Strategy ii:** Genomic structure screening.One of the strategies used by Abbott, Affinium, Anadys, Aventis, British Biotech, and GlaxoSmithKline to chemically optimize lead structures identified based on their target is to develop antibiotics based on their structure. Three-dimensional structures can be used to virtually explore complex databases through the use of docking techniques and in silico modeling methods [29]. In addition, the functions of basic proteins (proteins with unknown functions) can be determined. With genomic and structural data of target proteins, we can characterize the active sites of various pathogens, find potent and effective inhibitors in the body, and improve lead-optimization programs to increase potency and inhibitory activity and improve the antibacterial spectrum of the chemical lead chain.

**Strategy iii:** Whole-cell antibacterial screening.

Several pharmaceutical companies, including Abbott, Bayer, and GlaxoSmithKline, are working on discovering antibiotics that inhibit the growth of whole bacterial cells [30]. In this way, the number of hits on the target can be increased, and a greater number of targets for a pathogen’s physiological process or pathway can be tested [31]. Antibiotics discovered based on this method are the most marketable because they allow the biochemical function of the target to be known even if certain elements are unknown since they have the advantage of targeting more than one target, but their mode of action (MOA) is unknown [26,32,33,34].

## 4. Innovative Targets and Mechanisms of Action to Develop Potent Antimicrobials

Bacterial genome sequencing has made it possible to identify the genes necessary for bacterial cell survival, and these genes can be compared with other microbial species, helping to identify potential and common targets among a range of bacterial species [19]. The application of genomics has led to unprecedented advances in the identification of a large number of targets that have been validated and has provided an understanding of the emergence of drug resistance [5]. The target identified by genomics could be a receptor for the ligand in a bacterial molecule that has a specific function, be it an enzyme or a metabolic pathway. For this target, several conditions must be met: (i) the target has no counterpart in mammalian cells; (ii) the target must be present in human pathogens; and (iii) inhibition or elimination of the target results in the death of the bacterial cell or prevents it from causing disease. Then, we search for the lead molecule that can inhibit the target, e.g., inhibit the function of the metabolic pathway. Finally, all lead molecules are tested and then refined in a preclinical program [8]. So, the complete genome sequence of human pathogens enables more efficient research and helps research laboratories find important answers in functional genomics, thus contributing to the identification of innovative targets for antimicrobial drug development [35]. The use of genome sequencing to discover innovative targets for cell membranes and protein synthesis inhibitors is discussed below.

### 4.1. Cell Membrane Innovative Targets

Bacterial membranes consist of a group of highly regulated lipids and proteins. These membranes have an important role in multiple cellular functions, such as cell proliferation, migration, host cell adhesion, and secretion of virulence factors; thus they are attractive targets for antibiotic development [36]. Infection with Gram-negative bacteria is known to be one of the greatest challenges for drug developers because they are characterized by an outer membrane that prevents small antibiotics from reaching their target [37,38]. Lipid A represents the hydrophobic group of lipopolysaccharides (LPS) and forms the monolayer of the outer membranes of most Gram-negative bacteria [39,40]. In *E. coli*, lipid A is synthesized by a conserved metabolic pathway consisting of nine essential enzymes. The presence of lipid A in LPS is essential for bacterial growth and is one of the most important components of bacteria [41]. Therefore, the metabolic pathway of lipid-producing protein A is inhibited, resulting in damage to the bacterial cell membrane. To combat the problem of bacterial resistance to antibiotics, some necessary and unknown cellular metabolic pathways can be accessed. The use of automated high-throughput sequencing of genomic DNA, as well as the availability of whole genome sequencing of pathogens, has helped to identify the metabolic pathways required for bacterial cell membrane formation, which are innovative targets for new drug development [19,42]. 

Serral and coworkers are currently working to develop a curriculum that complements traditional wet-lab approaches, including genomics, structural bioinformatics, and cellular metabolic pathway analysis [43]. A series of inhibitors of bacterial membrane proteins have been proposed for Gram-negative bacteria [44]. On the other hand, Tn-Seq was used in *S. pneumoniae* interacting with 20 different antibiotics to study the genome scope and its connection to cellular processes. This is also used to determine the function of unknown function genes via observing the resulting interactions. This study focused on genes associated with the cell membrane to identify innovative targets. Based on the results of testing hundreds of isolates, it was determined that several genome-level changes in genes lead to a decrease in the sensitivity of bacteria to antibiotics, which is often associated with drug resistance. Accordingly, we can use the creation of a genome-wide atlas to identify susceptibility determinants and use specific interactions to redirect the strategies for identifying novel antibiotic targets [45].

#### 4.1.1. LpxC Inhibitors

LpxC (UDP-3-O-(R-3-hydroxymyristoyl)-N-acetylglucosamine deacetylase) is the major enzyme catalyzing the first step of lipid A biosynthesis in most Gram-negative species [46]. The absence of LPS in the outer membrane of Gram-negative organisms is considered to have a bactericidal effect. So, the absence of lipid A impedes the aggregation of LPS, which leads to bacterial cell death [47,48,49]. Therefore, the use of inhibitors against LpxC is an unprecedented strategy because it will have great efficacy against many human pathogens, including *Pseudomonas aeruginosa* and the *Enterobacteriaceae* family as shown on Table 1 [2]. 

LpxC inhibitors were first introduced in a medicinal chemistry program at the University of Washington (UW) and Chiron because the LpxC inhibitor was shown to stop the growth of *P. aeruginosa* [50]. In 2003, no less than 1200 new LpxC inhibitors were synthesized, most of which were active with MICs of < 1 µg/mL for both *P. aeruginosa* and *E. coli*. In addition, several compounds with MICs of 3 µg/mL or less are active against mouse models of systemic infection, with *P. aeruginosa* having an ED50 of 10 to 50 mg/kg and *E. coli* having an ED50 of 1.2 to 10 mg/kg [50]. On the other hand, ACHN-975 [51,52] from Achaogen and PF-5081090 [2] from Pfizer were the most advanced, effective compound, and complete microbiological descriptions of them were presented. In vitro, both showed effective bactericidal activity against both *P. aeruginosa* and *E. coli* with an MIC90 value of 1 µg/mL or less. Although both compounds were effective against a range of Gram-negative pathogens, they differed in their activity against cystic fibrosis pathogens. For example, PF -5081090 was observed to be effective against both *Burkholderia cepacia* and *Stenotrophomonas maltophilia*, whereas ACHN-975 showed no activity against these species [50]. The efflux pumps of these isolates differ in their substrate specificity, and PF-5081090 may be less sensitive to efflux by CF pathogens compared to ACHN-975 [2]. ACHN-975 is concentration-dependent against *P. aeruginosa*, while it is time-dependent against both *E. coli* and *Klebsiella pneumoniae* [51].

In addition, Tomaras et al. and coworkers in 2014 developed LpxC-4 (PF -5081090), which has a broad spectrum of activity for many pathogens and is highly effective in vivo against several strains of human pathogens. Standard static time-kill studies (STK) were performed with a range of different concentrations of LpxC-4 above and below the MIC against strains of *P. aeruginosa* and *K. pneumoniae*. LpxC-4 showed rapid bactericidal activity against *P. aeruginosa*, with limited regrowth at drug concentrations ≤2× the MIC (0.25 µg/mL). In addition, resistance frequency methods (FOR) were applied to previous strains to determine the ability of bacteria to develop resistance to LpxC inhibitors. Resistance frequencies of *P. aeruginosa* showed a very low probability of developing spontaneous resistance to LpxC-4, based on LpxC-4 concentrations above the MIC [2].

On the other hand, CHIR-090 is the most potent and active LpxC inhibitor discovered to date. It is an excellent antibiotic that strongly controls the growth of *E. coli* and *P. aeruginosa* and has the same efficacy as ciprofloxacin or tobramycin as shown on Table 1 [53,54]. CHIR-090 acts as a potent, fast-folding inhibitor of LpxC deacetylase from the hyperthermophilic Aquifex aeolicus [55]. CHIR-090 was tested in vitro, where it produced potent and slow inhibition of LpxC activities at only low nanomolar concentrations in several different bacteria, including *E. coli, H. pylori*, *Neisseria meningitidis*, and *P. aeruginosa*. However, CHIR-090 showed relatively weak competitive activity as an inhibitor of LpxC from *Rhizobium leguminosarum* [54,55].

#### 4.1.2. FabI Inhibitors

The type II bacterial fatty acid biosynthesis pathway (FAS-II) is highly conserved, making it an important target for the development of new antibiotics. The last step in each elongation cycle stimulates bacterial bioacidification (FabI), FabI isoforms FabL, FabK, FabV, and InhA, enoyl acyl carrier protein reductase (ENR) in many microorganisms [56]. Moreover, the components of FAS-II metabolism differ from the FAS-II multienzyme complex present in mammalian cells. Therefore, inhibition of FabI and its isoforms is expected to result in broad-spectrum antibiotic activity. Many efforts are being made to develop antibiotics with potent and effective inhibition of FabI [56]. On the other hand, FabI has been fully validated as a target in all *Staphylococcal* strains, making it an optimal target for novel antimicrobial agents targeting common pathogens such as *S. aureus* and MRSA [57].

Although several FabI inhibitors are known for high-throughput screening, most of them are not suitable for antibiotic development because they are not membrane-permeable, are volatile, and have a high mutational range [58]. There are a few FabI inhibitors that have shown good results in inhibiting FabI, and most of them are phenolic compounds. Meleagrin is an effective FabI inhibitor; it is isolated from Penicillium chrysogenum F717, a penicillin-producing species. The potency of inhibition of meleagrin was tested on both *E. coli* and *S. aureus* FabI and showed inhibition with IC50 of 33.2 and 40.1 μM, respectively. On the other hand, it was tested to determine whether meleagrin exerts a selective inhibitory effect on FabI. In tests with *S. pneumoniae* containing only Fabk, migraine *S. pneumoniae* did not inhibit FabK even in the presence of 200 μM, indicating the selectivity of meleagrin for Fab [59].

Meleagrin has chemically transformed into several derivatives, and whether the structural changes of meleagrin affected its activity in inhibiting FabI was investigated (Table 2). This was tested against *S. aureus* and *E. coli* FabI and bacterial growth. Compounds 5 and 6 modified by 9- OH and 14-NH groups showed a significant increase in their inhibitory activity for *S. aureus* and *E. coli* FabI. Compounds 2, 3, and 4 modified by 1-NH, 9- OH, and 14-NH groups did not change their activity, but compound 7 was found to have completely lost its antibacterial activity because it was brominated in the benzene ring [59].

A high throughput assay for FabI in *S. aureus* showed relatively weak inhibition with no antibacterial activity against *S. aureus*. David J. Payne and co-workers identified compound 4 [(E)-N-methyl-N-(2-methyl-1H-indol-3-ylmethyl)-3-(7-oxo-5,6,7,8)-tetrahydro-1,8-naphthyridin-3-yl)acrylamide] using iterative medicinal chemistry and X-ray crystal structure-based design. Compound 4 has astonishing antibacterial activity against *S. aureus*, resulting in the MIC of up to 90% of isolates being more than 500-fold lower than 9 antibiotics currently used against both *S. aureus* and *Staphylococcus epidermidis*. Compound 4 is 350 times more potent than the original lead compound. In addition, Compound 4 demonstrated significant in vivo inhibition of FabI in mouse models infected with *S. aureus* bacteria. After optimization, compound 4 proved to be even more effective in inhibiting FabI, resulting in its ability to inhibit FabI in multidrug-resistant strains of *S. aureus* and *S. epidermidis*. In addition, its activity was increased against microorganisms that utilise the FabI enzyme, such as *H. influenzae, E. coli*, and *Moraxella catarrhalis*. Of note, the ability of compound 4 to inhibit relatively weak FabK was observed, allowing it to exhibit antibacterial activity against both *S. pneumoniae* and *Enterococcus faecalis* [26].

### 4.2. Protein Synthesis Inhibitor as an Innovative Target

Protein synthesis plays an important role in controlling cell size, which is closely related to bacterial cell growth rate, so limiting protein synthesis by antibiotics will be deleterious to bacterial growth [60]. Protein synthesis inhibitors are one of the main classes of clinically useful antibacterials [61] due to the key roles of proteins in important biological processes, making them important targets for antibiotic development [62]. Genomics and genetics play a critical role in this process, as genomics helps test the target gene after it is important for cell growth and stability. The gene is then cloned, and the protein product is expressed and purified to test a variety of molecules to identify inhibitors of the target protein. In addition, genomic analysis allows the identification of basic metabolic pathways and key steps in protein synthesis, making them attractive targets for antimicrobial drug development [63,64]. Some of the innovative targets of antibiotics discovered by using genomics are discussed below.

#### 4.2.1. Peptide Deformylase Inhibitors

Peptide deformylase (PDF) belongs to the class of metalloenzymes and is the enzyme responsible for catalyzing the post-translational removal of one of the N-terminal methionine groups, the N-formyl group [65]. PDF is one of the best examples of protein synthesis inhibition. PDF is encoded by a def gene found in all bacteria, including *Mycoplasma* and *Chlamydia species*, but this gene has no counterpart in mammalian cells [10]. PDFs are important targets in the development of new antimicrobial agents. They are not only used as antimicrobial targets but also as targets for antimalarial and anticancer drugs after a similarity was discovered in the genetic sequence of PDF in parasites such as *Plasmodium falciparum* and humans [65].

Since PDF is an enzyme that is very important for bacterial growth, while mammals do not require it, it is a promising and selective target for the development of new antimicrobial agents [66]. Apfel and his partners provided experimental evidence for the efficacy of inhibition of the PDF: (1) Overexpression of PDF in bacterial species can inhibit bacterial growth, as has been shown in *E. coli*, *Streptococcus pneumoniae*, and *Haemophilus influenzae*. In contrast, a decrease in the expression of PDF in *S. pneumoniae* was shown to increase inhibitory activity. (2) Proteomic analysis revealed that in the presence of PDF inhibitors, many proteins shifted toward a lower pI value, which would be expected if these proteins still carried an N-formyl Met terminus. (3) *E. coli* should not be affected by PDF inhibitors if their growth conditions are independent of formylation and deformylation [67].

Studies conducted at the beginning of the 2000s discovered that marine bacterial strains can produce secondary metabolites with bactericidal activity because they have their metabolic pathway that produces metabolites with new structures and new activity. This is because they live in a very different environment than their counterparts on Earth [68]. *Bacillus subtilis* and its relatives such as *Bacillus amyloliquefaciens* and *Bacillus licheniformis* are the most commercially important strains because they are used to produce a group of secondary metabolites (e.g., amino acids, vitamins, and antibiotics) including polyketides [69]. Using bacterial genomics for these sequences, several potential secondary metabolites have been identified as effective antibiotics against multidrug resistance. For example, genomics has shown that about 8% of the genome sequence of *Bacillus* strains is destined for antibiotic production [70]. Surprisingly, strains capable of synthesizing secondary metabolites with broad-spectrum antibiotic activity can be identified by the presence of biosynthetic gene clusters (BGCs), including polyketide synthase (PKS) and nonribosomal peptide synthetase (NRPS), which encode many large multimodular polypeptides required for their synthesis [71].

Some secondary metabolites with antibacterial activity have been identified from the polyketide class, including macrolactins, macrocyclic lactones, and difficidin [72]. Polyketides are natural products with biological activity produced by microorganisms and belong to the classes known for their broad spectrum of activity. They are biosynthesized from acyl-CoA thioesters catalyzed by PKs [73,74]. Using genomics and modern techniques, Chakraborty Kajal and partners identified antibacterial secondary metabolites in *B. amyloliquefaciens* MTCC 12716 and predicted BGCs encoding target secondary metabolites. Three homologous members of the macrocyclic family were identified as bacvalactones (1–3): (1) bearing 13-Oethyl; (2) 15-O-furanyl-13-O-isobutyl-7-O-propyl-propanoate; and (3) 15-O-furanyl-13-O-isobutyl- 7-O-propyl-propanoate7,24-dimethyl fand were obtained using the bioactivity-guided purification technique [72]. 

The macrocyclic lactone components (1–3) showed potent biological activity against several opportunistic pathogens, including methicillin-resistant *Staphylococcus aureus* (MRSA), vancomycin-resistant *Enterococcus faecalis* (VREfs), and multidrug-resistant strains of *Pseudomonas aeruginosa* and *Klebsiella pneumonia* with MIC ≤ 3.0 μg/mL, while other standard antibiotics (ampicillin and chloramphenicol) had bioactivity only at concentrations greater than 6.25 mg/mL (Table 3). In addition, the activity of the macrocyclic lactone compounds (1–3) was confirmed using molecular modeling of silico against *S. aureus* PDF with all bacvalactones (1–3) and docking scores (≥9.70 kcal/mol) greater than those of the natural peptide deformylase inhibitors macrolactin N and actinonin, being 9.14 and 6.96 kcal/mol, respectively. Using genomic approaches in combination with current techniques, macrocyclic lactone compounds are effective against multidrug-resistant pathogens [72].

#### 4.2.2. Histidine Kinases Inhibitors

**Figure 3:**
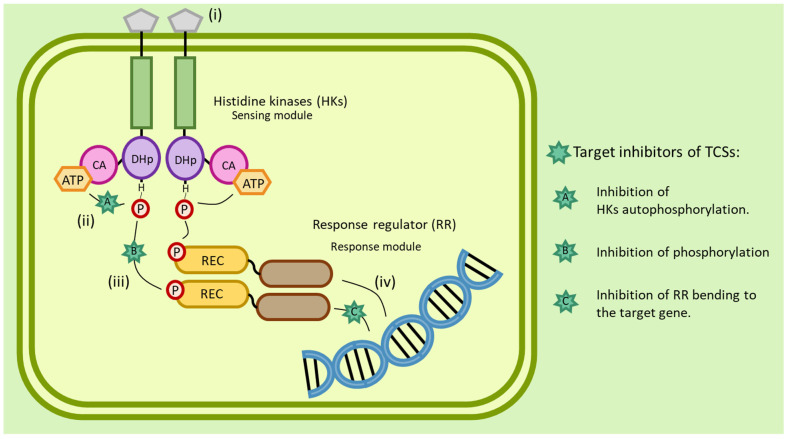
Overview of the bacterial TCS system and targets of inhibitors against TCS components. Signal transduction by TCS is initiated by stimulation of the sensing domain: (i) the CA domain binds with APT, and the HKs in the DHp domain are autophosphorylated; (ii) subsequently, the phosphoryl group is transferred to the Aps residue in the RR; (iii) a reaction that regulates the expression of target genes is activated; (iv) TCSs can control the gene expression of many important processes in bacteria.

Histidine kinases (HKs) function as part of two-component signal transduction systems. Bacterial two-component systems (TCS) consist of two major signaling proteins, histidine kinases (HKs) and the cytoplasmic response regulator (RR) protein as shown in Figure 3 [75,76]. Through these systems, important processes such as virulence, cell motility, secretion systems (SS), antibiotic resistance, and adaptation to environmental stress are regulated [6,75]. For example, histidine kinases of *E. coli* play a key role in sensing changes in the environment and contribute to adaptation and survival [77]. TCS are most commonly involved in the regulation of bacterial signal transduction [6]. They initiate autophosphorylation of HKs in the bacterial membrane, which is followed by phosphorus transfer of the phosphoryl group to the RR, which in most cases binds to bacterial DNA to allow expression of target genes [78]. There are tens to hundreds of different TCSs in bacteria [79]. The number of TCSs present in specific types of bacteria depends on the size of the bacterial genome and the range of environments in which the bacteria can live and grow [80]. The presence of TCSs and HKs is necessary for bacterial survival and growth. They are closed pangenomes, making them unique targets for antibacterial drugs with no toxicity to mammalian cells [6,81].

Using genomics and gene function analysis, researchers were able to describe many TCSs in different bacterial species. In the core genome of *S. aureus* strains, 17 TCSs were identified [82]. In addition, 29 HKs, 32RR, and 1 histidine-containing phosphotransfer domain (HPt) were identified in *E. coli* K12 by whole genome sequencing [83]. Sensor and effector domains and regulatory mechanisms are known to differ between different TCSs. Interestingly, the catalytic ATP-binding domain (CA) and the dimerization and histidine phosphorylation domains (DHp) are highly conserved in HKs. The genome sequences of the CA domains are similar in different HKs, suggesting that broad-spectrum antibiotics can be developed and would be a strategy to slow down the development of resistance [6,83].

Thiophene was developed based on the crystal structure of the ATP pocket of WalK. A series of thiophene derivatives were synthesized, and 8 compounds (Boibessot-6c, -6d, -6e, -6h, -6i, -6k, -6s, and -7c) were found to inhibit autophosphorylation of HKs WalK, PhoR, and ResE of *B. subtilis* in vitro, with IC50 ranges of 52.81–196.9, 1.63–122.6 and 20.3–243.9 μM, respectively. Both boibessot-6d and -6e showed antimicrobial activity against both Gram-negative and Gram-negative bacteria with MICs of 7–32 μg/mL, with boibessot-6d being slightly stronger than boibessot-6e. Both compounds had good activity against *S. aureus, S. pyogenes, Listeria monocytogenes, Salmonella enterica,* and *E. coli* with a MIC range of 10–32 μg/m, which is higher than against *B. subtilis* and *Bacillus anthracis* whose MIC is only 7 μg/mL. The researchers claimed that Boibessot-6d had a slower activity in cell lysis, with a duration of 9 h, but Boibessot-6e had a faster activity in cell lysis, so they could be used as bactericides [84].

The metabolites of *Streptomyces* species MK844-mF10 were studied to identify inhibitors of KHs targeting dimerisation and DHp domains. Waldiomycin was successfully isolated, which acts as an inhibitor of WalK and belongs to the angucycline antibiotics. Waldiomycin showed moderate activity against both *S. aureus* and *B. subtilis*, including methicillin-resistant strains with MICs of 8 μg/mL to 16 μg/mL. However, waldiomycin showed no activity against *Enterococcus faecalis*, *Mycobacterium smegmatis,* and Gram-negative bacteria [83]; qRT-PCR analyses of WalR regulon genes helped to show that waldiomycin inhibits the WalK/WalR system in *B. subtilis* and *S. aureus*, leading to inhibition of the expression of metabolic genes responsible for cell wall metabolism [85]. Several studies indicated that waldiomycin acts against Gram-negative bacteria by suppressing class I HKs such as *E. coli* QseC, EnvZ, and PhoQ with IC50s of 15.1, 22.4, and 12.5 µM [86]. 

#### 4.2.3. Aminoacyl-tRNA Synthetases Inhibitors

Several antibiotics that target the biosynthesis of proteins that are essential for bacterial cell growth have been introduced to the market. Among the innovative targets are aminoacyl-tRNA synthetases (AaRS). These are enzymes that convert amino acids into similar RNAs. These enzymes play an important role in the survival of pathogens. AaRS inhibitors include methionyl-tRNA synthetase (MetRS), isoleucyl-tRNA synthetase (IleRS), and phenylalanyl-tRNA synthetase (PheRS) inhibitors [87]. MetRS is a selective target because the type found in bacterial cells is distinctly different from that found in the mammalian cytoplasm. All previous and new selective MetRS inhibitors have been verified against a range of *Staphylococcus*, *Enterococcus*, and *Streptococcus* strains with MICs of ≤1.3 μg/mL. On the other hand, it was found that the activity of the inhibitors against Gram-negative bacteria was low because they contain a different type of MetRS enzyme [88]. Natural products that inhibit tRNA synthetases are effective, but there are many obstacles to their development as promising antimicrobial agents. However, the progress we are now seeing and the use of genomics technologies, high-throughput screening of complex libraries, novel inhibitor frameworks, and the identification of unknown functions of AaRS will provide a boost to the development of molecules for these targets. For example, comparison of genome sequences and structural analyses of different AaRS species in bacteria have helped to gather accurate information about structures that can be used as attractive targets [89].

Mupirocin is the only approved AaRS inhibitor that targets IleRS structures [90]. To date, the study of the genomes of antibiotic-producing Gram-negative bacteria has been the least explored. Nevertheless, it has been found that strains of *Pseudomonas fluorescens* can produce antagonistic substances. The use of mutational synthesis was proposed to discover new inhibitors, which increased the importance of genetic analysis of *P. fluorescens* strains. The BGCs required for mupirocin were discovered using a series of transposon insertion mutants. Mutations of mupirocin by cloning and sequencing BGCs helped to suggest plausible biosynthetic pathways. Finally, mupirocin production was improved by optimizing the traditional *Pseudomonas* strain NCIMB10586, which is one of the sub-strains of *P. fluorescens* [91].

Although mupirocin is effective in inhibiting AaRS and is approved for clinical use, methicillin-resistant *Staphylococcus aureus* (MRSA) is increasingly developing resistance to this agent. A new AaRS inhibitor called Batumici has been identified. Computational molecular docking and whole genome analysis of Batumici producers (*Pseudomonas batumici* UCM B-321) were used to identify potential molecular targets of Batumici. Previously, it had been assumed that the ideal target for Batumici was the trans-enoyl-CoA reductase FabI, but this was rejected. The structures of the leucine-tRNA synthetases in the genome of *P. batumici* showed that this protein is the optimal target for Batumici. The biosynthetic operon of Batumici was found to contain 28 genes that arose by horizontal gene transfer. Remarkably, Batumici has a broader ability to inhibit AaRS compared to mupirocin. Furthermore, MRSA cannot develop resistance to Batumici due to its acquired resistance to mupirocin [92].

**Figure 4:**
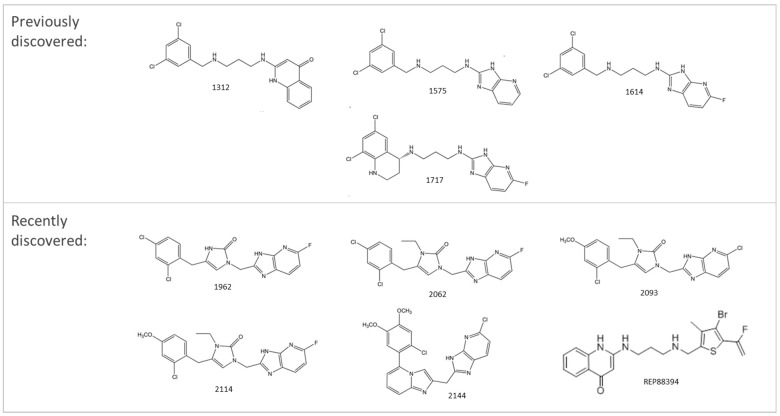
Chemical structure of MetRS inhibitors. This chemical structure was prepared based on Faghhih et al., (2017) [88]; Shibata et al., (2011) [93]; and Zhanz et al., (2016) [94].

Several compounds that had been previously studied were reported, namely 1312, 1575, 1614, and 1717 [93,94], and the newly published compounds were 1962, 2062, 2093, 2114, and 2144 as shown in Figure 4. All of these MetRS inhibitors showed effective antibacterial activity against various Gram-negative bacterial strains, and there was no inhibitory activity against Gram-negative bacteria, especially *P. aeruginosa* and *E. coli.* Specifically, the efficacy of the compounds was measured against several bacterial strains, including *S. aureus*, methicillin-susceptible S. aureus (MSSA), MRSA, vancomycin-intermediate S. aureus (VISA), *Staphylococcus epidermidis, Enterococcus faecalis, Enterococcus faecium,* vancomycin-susceptible enterococci (VSE), and VRE, and MICs of < 0.3 μg/mL were measured. It is worth noting that the compounds with the lowest MIC values were 1717, 2093, and 2144, which had more than a 10-fold activity against a range of strains compared with vancomycin [88].

#### 4.2.4. Riboswitches as Target

Riboswitches are non-coding RNA gene control structures usually found at the 5′ end of messenger ribonucleic acid (mRNA). Riboswitches regulate and control the translation and transcription processes of essential metabolites by selectively binding to small molecules or ion ligands. These essential metabolites that without them cells cannot function [95,96,97]. The various riboswitch mechanisms involve two functional domains, an aptamer, and an expression platform. The aptamer selectively binds to the targeted small molecule, and the expression platform responds to the binding of the target molecule and regulates and controls gene expression in an allosteric manner. The control of gene expression can be either turned off or activated depending on the function of the small molecule [98]. Currently, 28 riboswitch classes are known, and the number of newly discovered classes is approximately 3 riboswitches per year [96,98]. Riboswitches are distributed across more than 6000 bacteria, including human pathogenic bacteria [96]. Therefore, riboswitches are considered a suitable target for broad-spectrum antibiotics. Riboswitches that are only present in the eukaryotic including algae, fungi, and plants are the thiamine pyrophosphate (TPP) riboswitch, which is the most prevalent riboswitch. On the other hand, the most prevalent riboswitches in the pathogenic bacteria are flavin mononucleotide (FMN), cobalamin, glutamine-fructose-6-phosphate (glmS), lysine, S-adenosyl methionine-I (SAM-I), and glycine riboswitches [96,99].

Methods for discovering new classes of riboswitches were developed by understanding the data and genomic sequences that indicated that riboswitches were already present in bacterial derivatives of genes. The first riboswitches observed were the most abundant riboswitches. For example, each of the lysine, flavin mononucleotide (FMN), and adenosylcobalamin (AdoCbl) classes is the most abundant riboswitch among the 10 best-known classes [96,100].

The most common mechanisms of action of gene control mediated by riboswitches include direct and indirect methods. The direct method is to regulate translation, while the indirect method is to inhibit protein production by altering the mRNA sequence or messenger RNA [100].

Flavin mononucleotide (FMN) is found in 41 pathogenic bacteria, including *A. baumannii, P. aeruginosa, Enterobacteriaceae, E. faecium, S. aureus, S. pneumoniae, H. influenzae,* and *Shigella* spp. FMN riboswitch regulates the expression of genes responsible for riboflavin biosynthesis and transport. As a precursor, a coenzyme of FMN and flavin adenine dinucleotide plays an essential role in oxidative phosphorylation, β-oxidation, and the Krebs cycle [96].

Several factors make the FMN riboswitch one of the most important targets for broad-spectrum antimicrobials: (i) its important role in regulating the physiology of bacteria, (ii) a highly conserved binding domain, (iii) it has no analogs in human cells, and (iv) it is abundant in the cells of pathogenic bacteria. The FMN riboswitch is the main target for an antibacterial component called roseoflavin. Roseoflavin is a natural analog of riboflavin synthesized by *Streptomyces davawensis.* Roseoflavin can inhibit the growth of bacteria possessing its phosphorylated form (RoFMN), including *B. subtilis, E. coli, and L. monocytogenes*, by targeting the FMN riboswitch [96].

## 5. Developing Targeted Antivirulence Therapeutics

Virulence factors are molecules that enable bacteria to infect host cells and help the bacteria to multiply and colonize the host at the cellular level by eliminating or bypassing host defenses. Virulence factors can be secretory via the secretory system (SS), membrane-associated, and cytoplasmic. Secretory virulence factors support host cell attack and destruction. Membrane-associated virulence factors attach to host cells and evade the host immune system. Cytosolic virulence factors serve to adapt bacteria to their environment. Virulence factors are therefore thought to play a role in causing infections, so their inactivation helps prevent bacteria from causing infections [101,102]. Virulence-arresting drugs (VADs) are a class of antimicrobials that disarm rather than kill the bacteria, causing them to lose their ability to cause disease in host cells [103]. The VAD has the least chance of developing resistance because it relies on preventing bacteria from causing disease, so it would not pose a significant burden in developing drug resistance [104]. The use of genomics to identify primary genomes as targets for VAD offers the opportunity to improve the use of these expensive drugs, thus supporting the treatment of infectious diseases through the rational use of VAD [103].

Genomic approaches can be used to test the accuracy of VADs in different types of pathogens by identifying the genes in different types of bacteria that are responsible for their unique phenotype (i.e., virulence) and thus provide innovative targets for different classes of VADs [105]. We can divide the genes responsible for virulence into two categories: (i) genes responsible for cell death in the host and (ii) genes responsible for virulence factors (VFs) that occupy host cells and camouflage the host immune system. Thus, routine genome sequencing of pathogenic bacteria can be used to identify targets for VADs and determine whether there is a possibility that resistant mutations to VADs will develop. Because VADs can prevent bacteria from causing infection without killing them, there is a possibility that resistant mutations to VADs will develop [106,107]. For example, *P. aeruginosa* strains have shown resistance in vitro to furanone C-30, a type of QS -inhibited VADs, due to silencing mutations in the *mexR* gene [106]. This gene is responsible for the negative regulation of efflux pumps and is also involved in the release of the auto-stimulatory protein C12-HSL [108]. However, it has not yet been fully documented as resistance testing for VADs is yet to be carried out.

Metapopulations are used to describe the dynamics of organisms of the same species that are spatially separated, the stability of populations, and the coexistence of species when those species interact at some level [109,110]. Since the genome sequence differs between bacterial metapopulations, this may lead to the masking of target proteins in minority bacterial populations, as in the known pathogens *Salmonella typhimurium* and *S. pneumoniae* [111,112]. Therefore, the study of the target proteins of VADs in different bacterial strains may reveal different therapeutic efficacy of drugs, as this change in the efficacy of VADs may be due to the absence of coding genes or/and a difference in their genomic sequence. For example, the *lasI/lasR* gene in *P. aeruginosa* and the *comD* gene in *S. pneumoniae*, which are targeted by the inhibitor QS, were found to account for only 94.5 and 57.8%, respectively, in other strains [103]. Some of the methods used to develop targets for VADs using bacterial genome sequencing are presented below.

(A) VADs anti-adhesion:

The binding proteins on the surface of the bacterial membrane form the fundamental part and the first step to disease development, as these proteins bind to specific receptors on the surface of host cells; therefore, preventing this binding may be an appropriate strategy. This is achieved by making the binding proteins targets for VADs, which slows host cell invasion and provides time for the immune system to recognize their presence and initiate an immune response [113,114,115]. For example, licoflovanol, a small molecule that inhibits adhesion by inhibiting the secretion of effector proteins secreted by bacterial secretion systems (SS) in *Salmonella* pathogenicity island 1 (SPI-1), is used by regulating the transcription of *sicA/invF* genes [114,116,117].

(B) VADs Quorum Sensing Inhibitors:

Disrupting the cell-to-cell communication system using small chemical signaling molecules known as quorum sensing (QS) is one of the best ways to control virulence. These small signaling molecules activate cellular processes that promote bacterial community survival. These cellular processes affect both VF, biofilm formation, and the formation of antimicrobial resistance. Therefore, inhibition of QS results in a decrease or reduction of these signaling molecules and thus affects all cellular processes at the bacterial community level [118]. QS inhibition can be accomplished in several ways, all of which lead to the same result, namely the disruption of cellular processes. These methods consist of preventing the synthesis of QS signaling molecules, inactivating QS signaling molecules, or disrupting their receptors so that recognition of QS signaling molecules is prevented [119,120]. In *P. aeruginosa*, the QS system contains two N-acyl homoserine lactone (AHL) regulatory circuits that are las and rhl genes and use hierarchically linked alkyl 4-quinolones (AQs) in non-AHL-mediated QS signaling pathways [119]. Indeed, several QS -inhibitory factors have been found to disrupt key QS signaling pathways, block the synthesis of AHL-sensitive molecules, or prevent the detection of AHL by disrupting its receptors, but they are still under clinical investigation [121].

## 6. Future Strategies in Discovering Innovative Targets for Novel Antibiotics

**Figure 5:**
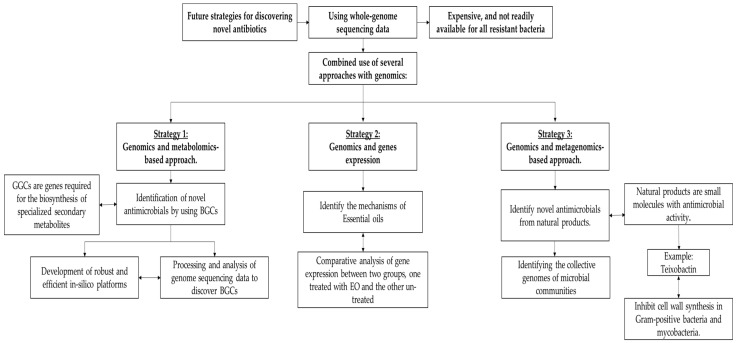
Combination of using several approaches with genomics for discovering novel antibiotics.

The techniques used in whole genome sequencing are gaining traction over conventional techniques. Today, various strategies are being pursued to discover innovative targets using whole-genome sequencing data. However, the use of genome sequencing is expensive, and not readily available for all mutants of drug-resistant bacteria in health centers [63]. Many believed that the use of whole genomics would open the doors to the discovery of many promising antimicrobials with innovative mechanisms and targets, and although this is true to some extent, in terms of discovering innovative targets, it is difficult to identify the active site and/or mechanism experimentally [63]. Therefore, researchers have considered the combined use of several approaches in addition to genomics, including synthetic biology, metagenomics, and metabolomics, to find innovative targets and novel antimicrobial agents as shown in Figure 5 [122].

### 6.1. Identification of Novel Antimicrobial by Using BGCs

Genomic analyses have shown that microorganisms are capable of producing more metabolites than suggested by classical biomechanical screens [123], raising the ceiling for antimicrobial discovery through genomics and metabolomics-based approach. The provision of secondary metabolic pathways and the advent of bacterial genome sequencing contributed to the identification of many secondary metabolite biosynthesis gene clusters (BGCs) in bacteria that had not previously been observed in the laboratory. Identification of BGCs is one of the most important strategies in bioprospecting for new antibiotics. BGCs are defined as a mechanism required for the biosynthesis of specialized metabolites, and they consist of two or more contiguous genes [124,125]. These genes encode proteins involved in the production of these secondary metabolites, and these genes encode regulatory elements, resistance development factors, or transport proteins [124]. Although these secondary metabolites are not essential for bacterial cell survival and growth, they play important roles in pathogenesis, virulence, and environmental adaptation [126,127]. In addition, the identification of gene clusters responsible for antibiotic biosynthesis facilitates the development of new compounds and may allow the production of a metabolite through heterogeneous expression [124].

Since the first discovery of secondary metabolites by David Hopwood and his collaborators using whole genome sequencing of *Streptomyces coelicolor* [128], genomics has become the optimal strategy for accessing secondary metabolites, as the study of the bacterial genome has revealed large biosynthetic and undiscovered capabilities. In addition, analytical chemistry methods used to discover several compounds have been shown to lag behind the putative chemical entities discovered through genomics [129]. Currently, the greatest challenge is the processing and analysis of genome sequencing data to discover BGCs and relate them to the chemical structures of known secondary metabolites or newly suspected chemical entities. This has resulted in an urgent need to manipulate and screen genomic sequences, which has led to the promotion and development of robust and efficient in silico platforms, including antiSMASH 4.0 and PRISM 3, which primarily use bacterial genomes to discover BGCs and predict the structures of secondary metabolites that encode from these genes [130,131].

### 6.2. Using Genomics to Identify the Mechanisms of Essential Oils

Essential oils (EO) are one of the natural components that have an antimicrobial effect. EO is defined as secondary metabolites consisting of a complex mixture of compounds produced by aromatic plants. Previous studies have demonstrated the efficacy of EO as biological agents, as they have antimicrobial, antioxidant, antitumor, and anti-inflammatory effects in vitro and in vivo [132]. However, detailed information about the mechanism of action in inhibiting the bacterial membrane is not yet known. Thanks to new techniques and strategies, it has been possible to understand the mechanism of the antimicrobial action of essential oils. Genomics is helping to elucidate the properties of the mechanisms of antimicrobial action in essential oils. The main strategy here was to assess the genetic changes in disease-causing bacteria through a comparative analysis of gene expression between two groups, one treated with EO and the other untreated. In essential oil-treated *Campylobacter jejuni*, genes involved in disease pathogenesis, including metabolic processes, stress response, and transcriptional regulation, were examined and found to be an enhanced expression of genes responsible for oxidative stress. For example, 2D- SDS PAGE coupled with LC-MS /MS was used to compare *C. jejuni* with untreated peppermint essential oil. After treatment of *C. jejuni* with peppermint essential oil, the expression of oxidative stress-related genes dps, sodB, and katA increased. Similarly, the effect of oregano essential oil on *Salmonella enteritidis* was confirmed as several proteins related to oxidative stress (clpB, htpG, luxS, toxic shock protein, and USP) were observed in oregano essential-oil-treated *S. enteritidis* cells showing an increase in their expression based on the results of the assay using 2D- SDS PAGE and LC-MS /MS [133].

### 6.3. Using Genomics and Metagenomics to Identify Novel Antimicrobial Molecules from Microbial Communities

A group of researchers selected another group of antibiotics from various sources, including plants, animals, and microorganisms to study their efficacy as antimicrobial agents. Among these groups, the microbial community proved to be the most effective because it is the source of many small active antimicrobial molecules that facilitate survival in a competitive microbial environment [134,135]. Therefore, we need to study microbial complexes to access the active natural products that can be used as new antibiotics. In this context, neither culture-based nor chemical study approaches can be used to study the microbial complex because less than 1% of environmental bacteria can be cultured in the laboratory using standard methods [136]. Genomics was used in combination with metagenomics methodology to screen these compounds, identifying several small molecules with antimicrobial activity [137].

Metagenomics is known as a powerful, culture-independent technique that contributes significantly as a way to identify the collective genomes of bacterial groups living in natural habitats to find new antibiotic producers [123]. Recently, this approach was used to discover a new compound called teixobactin, which was extracted from soil. Teixobactin binds to the precursors of peptidoglycan and teichoic acid, resulting in the inhibition of cell wall synthesis in Gram-positive bacteria and mycobacteria [138]. In environmental bacteria, a large number of antibiotic biosynthetic gene clusters are not expressed and are therefore referred to as silent BGCs. Drugs are naturally derived from small portions of the main expressed BGCs, and the way silent BGCs are activated to produce new metabolites will greatly improve the discovery of new drugs [139]. It is impressive that old antibiotics can be used to find new ones by stimulating the expression of silent BGCs. However, transforming a natural product into an antimicrobial agent that can be used to treat bacterial infections remains difficult [123,140].

## 7. Conclusions

From this review, the discovery of bacterial genome sequences will enable the discovery and development of several antibiotics with innovative targets and mechanisms of action. In addition, this will help to take steps toward a definitive solution to the problem of the development of drug resistance [141]. Knowledge of the genome sequence of bacteria has made it possible to identify essential targets for bacterial growth and survival. As mentioned earlier, LpxC, PDF, AaRS, and FabI, which stimulate important biological processes, are attractive targets. On the other hand, it is possible to target the centers of signal transduction that are important for bacterial survival [19]. Thus, TCSs and HKs have been used as targets for antibiotic development. Genome sequencing has paved the way for the development of antibiotics against multidrug-resistant bacteria. The availability of new technologies and strategies for the development of new antibiotics is based on knowledge of the genome sequence of bacteria [142].

## Figures and Tables

**Table 1:** Microbiological assessment of activity of the novel LpxC inhibitors in vitro. This table was created based on CHIR-090 and LpxC-4 information from Tomaras et al., (2014) [2], and ACHN-975 information from Krause et al., (2019) [51]. NT: not tested; ND: not determined; IC50: half-maximal inhibitory concentration; MIC90: minimum inhibitory concentration of 90% of isolates.

LpxC Inhibitors	PF-5081090	ACHN-975	CHIR-090
Chemical structure	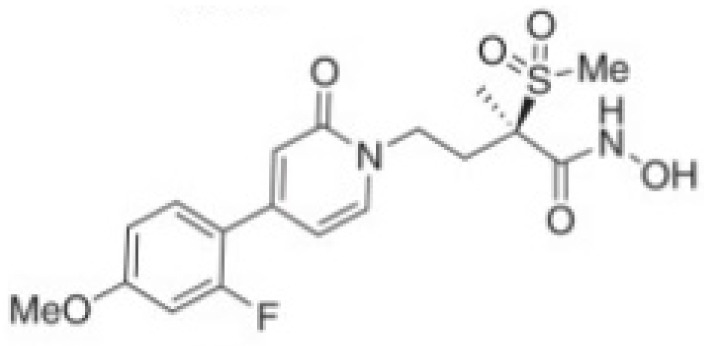	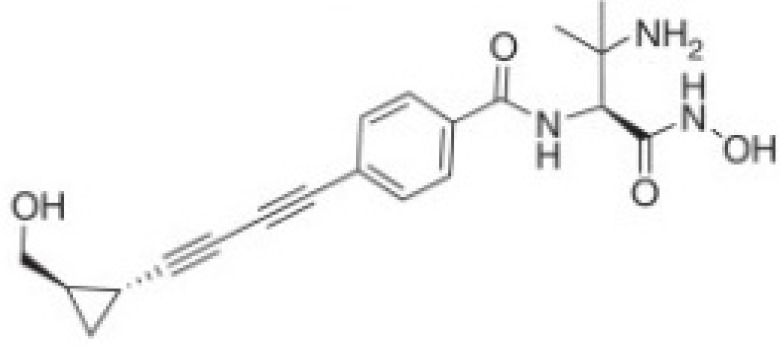	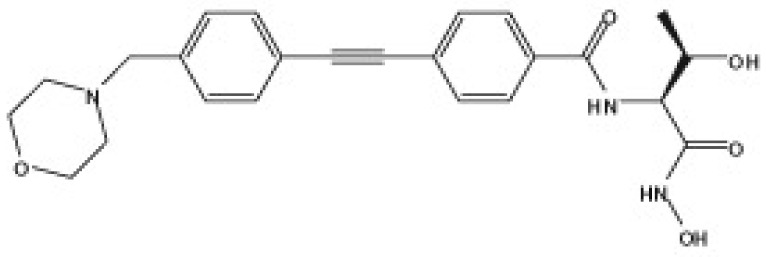
IC50 (nM)			
*P. aeruginosa* (138)	1.1	0.05	<2.1
*P. aeruginosa* PAO1 WTe	1.1	0.05	<2.1
*P. aeruginosa* PAO1 M62R	2.1	0.5	NT
*K. pneumoniae* (98)	0.069	ND	NT
*E. coli* (79)	NT	ND	NT
*Enterobacter spp.* (52)g	NT	ND	NT
*Acinetobacter baumannii* (31)	183	ND	NT
*Burkholderia cepacia* (30)	NT	ND	NT
*Stenotrophomonas maltophilia* (30)	NT	ND	NT
MIC90 (μg/mL)			
*P. aeruginosa* (138)	1	ND	4
*P. aeruginosa* PAO1 WTe	0.5	0.5	1
*P. aeruginosa* PAO1 M62R	0,5	0.5	NT
*K. pneumoniae*	1	2	NT
*E. coli*	0.25	0.5	0.25
*Enterobacter spp.*	0.25	ND	0.5
*Acinetobacter baumannii* (31)	>64	>64	>64
*Burkholderia cepacia* (30)	0.5	16	>64
*Stenotrophomonas maltophilia* (30)	2	>16	>64

**Table 2:** Inhibition activity of meleagrin and its chemically prepared derivatives. This table was prepared based on information from Zheng et al., (2013) [59].

FabI inhibitors	Chemical structure	MIC (μg/mL)		IC50 (μM)
		*S. aureus* RN4220	*E. coli* KCTC 1924	[^14^C] acetate incorporation
Meleagrin	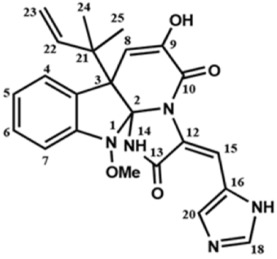	64	32	64
Compound 2	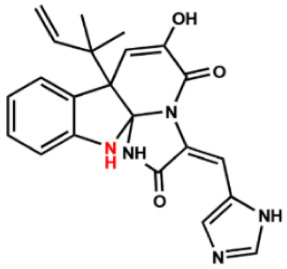	64	64	64
Compound 3	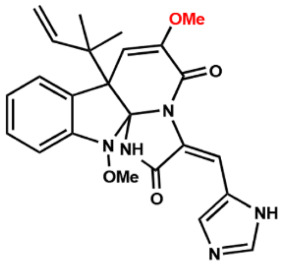	64	32	64
Compound 4	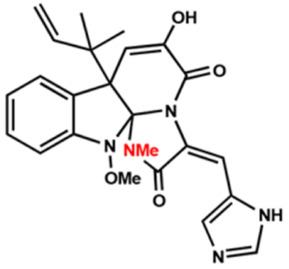	64	32	64
Compound 5	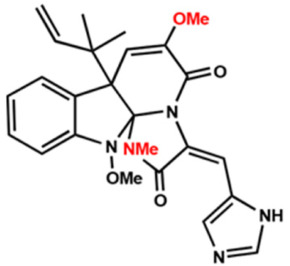	16	8	16
Compound 6	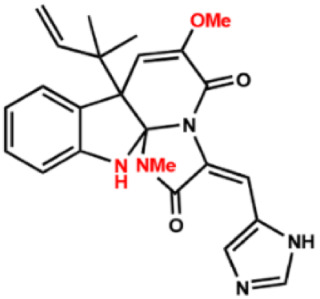	16	8	16
Compound 7	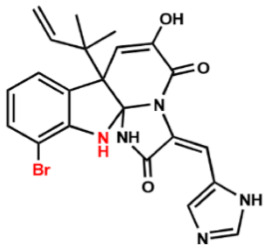	>128	>128	>128

**Table 3:** Comparison between the bioactivity of bacvalactones (1–3) and standard antibiotics. This table was prepared based on information from Kajal et al. [72]. ^a–c^ The column-wise value with superscripts indicates a significant difference (*p* < 0.05), which is implied for the statistical analysis of the data.

Antibiotics	Pathogens	Zone of Inhibition (mm)	MIC (μg/mL)
(1)	MRSA	21.00 ^a^ ± 0.05	3.00
	VREfs	22.00 ^a^ ± 0.02	5.00
	*Pseudomonas aeruginosa*	17.00 ^b^ ± 0.03	3.17
	*Klebsiella pneumonia*	19.00 ^b^ ± 0.03	5.00
(2)	MRSA	22.00 ^a^ ± 0.01	3.12
	VREfs	25.00 ^b^ ± 0.04	1.50
	*Pseudomonas aeruginosa*	23.00 ^a^ ± 0.02	3.00
	*Klebsiella pneumonia*	26.00 ^b^ ± 0.02	3.00
(3)	MRSA	30.00 ^a^ ± 0.01	1.50
	VREfs	28.00 ^a^ ± 0.04	3.00
	*Pseudomonas aeruginosa*	25.00 ^b^ ± 0.02	1.50
	*Klebsiella pneumonia*	31.00 ^a^ ± 0.02	1.50
Ampicillin	MRSA	10.00 ^a^ ± 0.02	12.50
	VREfs	13.00 ^b^ ± 0.04	25.00
	*Pseudomonas aeruginosa*	7.00 ^c^ ± 0.01	12.50
	*Klebsiella pneumonia*	7.00 ^c^ ± 0.02	12.50
Chloramphenicol	MRSA	14.00 ^a^ ± 0.02	6.25
	VREfs	13.00 ^b^ ± 0.01	6.25
	*Pseudomonas aeruginosa*	11.00 ^a^ ± 0.02	16.00
	*Klebsiella pneumonia*	7.00 ^c^ ± 0.04	12.50

## Data Availability

Not applicable.
